# Ti/Au Cathode for Electronic transport material-free organic-inorganic hybrid perovskite solar cells

**DOI:** 10.1038/srep39132

**Published:** 2016-12-20

**Authors:** Tongfei Shi, Jian Chen, Jianqiang Zheng, Xinhua Li, Bukang Zhou, Huaxiang Cao, Yuqi Wang

**Affiliations:** 1 Key Laboratory of Materials Physics, Institute of Solid State Physics, Chinese Academy of Sciences, Hefei, 230031, P. R. China

## Abstract

We have fabricated organic-inorganic hybrid perovskite solar cell that uses a Ti/Au multilayer as cathode and does not use electron transport materials, and achieved the highest power conversion efficiency close to 13% with high reproducibility and hysteresis-free photocurrent curves. Our cell has a Schottky planar heterojunction structure (ITO/PEDOT:PSS/perovskite/Ti/Au), in which the Ti insertion layer isolate the perovskite and Au layers, thus proving good contact between the Au and perovskite and increasing the cells’ shunt resistance greatly. Moreover, the Ti/Au cathode in direct contact with hybrid perovskite showed no reaction for a long-term exposure to the air, and can provide sufficient protection and avoid the perovskite and PEDOT:PSS layers contact with moisture. Hence, the Ti/Au based devices retain about 70% of their original efficiency after 300 h storage in the ambient environment.

Organic-inorganic hybrid perovskite materials (MAPbX_3_, X = halogen), which are composed of Earth-abundant materials and can be deposited by low-temperature solution methods, have attracted much attention for fabricating low-cost solar cells^1^,^2^, In the past six years, the power conversion efficiency (PCE) of perovskite solar cells (PSCs) has surged from 3.8% to over 23%^3^,^4^. Almost all kinds of PSCs, including normal structures (FTO/TiO_2_/MAPbI_3_/spiro-OMeTAD/Au) and inverted structures (ITO/poly(3,4-ethylenedioxythiophene):ploy(styrenesulfonate)(PEDOT:PSS)/MAPbI_3_/[6,6]-phenyl-C_61_-butyric acid methyl ester (PCBM)/C_60_/BCP/Au), are required to use the organic conductors, such as spiro-OMeTAD, PCBM and C_60_^5^,^6^,^7^. However, these organic materials are expensive, complicate the device fabrication process^6^, and more severely, they can limit the long-term stability of the devices^8^.

It is highly desired hope to find high-performance device structures without these organic conductors. At present, hole transport material (HTM)-free PSC is the most commonly used structure, which discard spiro-OMeTAD in the normal structures, with the highest PCE of about 12.8%^9^,^10^,^11^. Due to the nearly identical work functions of PEDOT:PSS and Au electrodes, the direct contact between Au and MAPbI_3_ in the inverted structures is generally considered to be unfavorable for charge collection. Hence, electronic transport multilayer, such as PCBM/C_60_/BCP, are often used to improve the devices performances^6^. Recently, Huang’s group fabricated electronic transport material (ETM)-free PSCs using Au as cathode, which provided a new Schottky type PSC structure^8^,^12^. At present, its PCE is below 8% and significantly less than that of HTM-free PSCs^12^, with large photocurrent hysteresis and lighting-soaking effects^8^,^12^. Moreover, the common metal cathodes, such as Ag, Al and Au strongly react with hybrid perovskite, which is one of the important reasons for the poor stability of PSCs^12^,^13^. Even, Cu in direct contact with perovskite exposure to the air should also be reacted^13^. Therefore, it is urgent to find new cathode materials to improve the performances of the ETM-free PSCs.

In this work, we report a new kind of high performance ETM-free PSCs with a maximum PCE close to 13%. Specifically, the ITO/PEDOT:PSS/MAPbI_3_/Ti/Au devices have been fabricated with Ti/Au multilayer as metal cathode, as shown in Fig. 1(a). The insertion of Ti layers can effectively improve the wettablity of Au and reduce charge traps at the pervoskite surface, Leading to the devices also show high repeatability and hysteresis-free photocurrent curves of the devices. The devices have a surprisingly high PCE of 9.2% after 300 h storage in the ambient environment, due to the complete isolation from moisture by the high stable Ti/Au cathode in the air.

Figure 1(b) shows the photocurrent curves of the ITO/PEDOT:PSS/MAPbI_3_/Ti/Au(80 nm) devices with different thickness of the Ti layers under 1.5 sun illumination. The photovoltaic parameters of each device are given in Table 1. It can be found that in the device with Au cathode, the PCE is only 6.3% with poor fill factor (*FF*, ~ 43%) and small open circuit voltage (*V*_*oc*_, 0.65 V). After the insertion of Ti layer (5 nm), an obvious PCE enhancement is observed, with the *FF, V*_*oc*_ and short circuit current (*J*_*sc*_) elevated to ~51%, 0.89 V and 23.64 mA/cm^2^, respectively. When the Ti film thickness raises to 10 nm, the highest PCE approaching 13% is achieved with the J_sc_ up to 24.38 mA/cm^2^. It demonstrates that the Ti/Au multilayer can effectively improve the device performance.

Energy band alignment of the device is shown in Fig. 1(c). The work functions of PEDOT:PSS (−5.3 eV) and Au (−5.1 eV) are both close to the valence band edge of MAPbI_3_ (−5.4 eV), which causes the carriers recombination at the electrodes and limits the charge collection. This is the main reason for the poor photovoltaic parameters of the ITO/PEDOT:PSS/MAPbI_3_/Au devices. In contrast, inserting a layer of Ti thin film can effectively raise the voltage and block the holes without affecting the electrons collection of cathodes, sinceA the work function of Ti is higher than that of PEDOT:PSS by about 1 eV and slightly lower than the conduction band edge of MAPbI_3_. Accordingly, the maximum *V*_*oc*_ and *J*_*sc*_ of the ITO/PEDOT:PSS/MAPbI_3_/Ti/Au devices have reached 0.89 V and 24.38 mA/cm^2^ (Table 1), close to those of the devices with ETM multilayer films^6^,^14^,^15^.

*The high repeatability of the ITO/PEDOT:PSS/MAPbI*_*3*_*/Ti(10 nm)/Au(80 nm) devices is shown in* Fig. 1(d–g)*. Photovoltaic parameters were gathered from 24 cells, that yielded small standard deviation, leading to averaged J*_*sc*_ *of 21.96* ± *1.42 mA/cm*^2^*, V*_*oc*_ *of 0.82* ± *0.04 V, FF of 50.27* ± *4.62% and PCE of 10.23* ± *1.21%. Moreover, the J-V curves of ITO/PEDOT:PSS/MAPbI*_*3*_*/Ti(10 nm)/Au device with increased and decreased bias at a scan rate of 0.05 V/s are shown in* Fig. 1(h). *Xiao et al. have demonstrated that the ITO/PEDOT:PSS/MAPbI*_*3*_*/Au devices should have a large hysteresis*^8^*. To our delight, there are only negligible changes in our sample’s photocurrent density with reverse and forward scan, even better than the cells with ETM (PCBM/C*_*60*_*/BCP)*^6^*. Therefore, the Ti insertion layers have not only improved significantly the ETM-free device PCE, but also achieved high repeatability and hysteresis-free photocurrent curves.*

It is worthy of note that the device performance has suddenly decayed when the Ti insertion layer thickness reaches 20 nm (Fig. 1(a) and Table 1). The PCE was less than 5% and the FF was close to 30%, which was even lower than those of the device using Au cathode. The reason will be discussed later.

Series resistor (R_s_) and shunt resistor (R_sh_) of the ETM-free devices with different cathodes (Au(80 nm), Ti (10 nm)/Au(80 nm) and Ti(60 nm)) have been also measured, and shown in Supplementary Figure S1. In the devices with Au, Ti/Au and Ti cathodes, the R_s_ are 1.39 Ω/cm^2^, 8.94 Ω/cm^2^ and 122.26 Ω/cm^2^and the R_sh_ are 68.31 Ω/cm^2^, 6409.8 Ω/cm^2^ and 23558.4 Ω/cm^2^, respectively. This indicates that the R_sh_ can be improved by two orders of magnitude with a proper thickness (~10 nm) of the Ti films, while the influence of the R_s_ is limited. As an indeal solar cell should have a small R_s_ and a larger R_sh_, the giant R_sh_ is beneficial for the devices to obtain excellent performance. However, with the increase of the Ti films thickness, the R_s_ increases rapidly. When the Ti cathode thickness reaches 60 nm, the R_s_ is also increased by 100 times. Because of increase of the internal resistance, the photovoltaic parameters of the cell with 20 nm Ti insertion layer have been greatly reduced and even less than the device with Au cathode.

*Scanning electron microscopy (SEM) images for MAPbI*_*3*_*/Au, MAPbI*_*3*_*/Ti/Au and MAPbI*_*3*_ *films are shown in* Fig. 2*, respectively. In consistence with other works*^16^,^17^*, the MAPbI*_*3*_ *was a polycrystalline film composed of grains with size ~300 nm. From the* Fig. 2(b) and (c)*, the Au films (10 nm and 20 nm) were also composed of nanoparticles with a pin-hole free surface. Moreover, from the cross section SEM shown in the* Fig. 2(d)*, it can be found that there is large density of voids at the interface between the Au and MAPbI*_*3*_ *films. These indicate that Au metal has a poor wettability on the surface of MAPbI*_*3*_*, and cannot form a uniform thin film. However, after the deposition of Ti and Au on MAPbI*_*3*_ *in turn, a firm and compact film is obtained as shown in* Fig. 2(e) and (f)*, which suggests that the Ti insertion layer can improve the wettability of Au on the surface of MAPbI*_*3*_*. In other words, the Ti layers can help to form a good contact between the MAPbI*_*3*_ *films and metal Au electrode, which can improve the charge collection and eliminate the void-inducing the charge accumulation. This is also one of the important reasons for the significant improvement in the related device performances, such as J*_*sc*_ *and FF.*

*Many works have reported the existence of a large density of charge traps in MAPbI*_*3*_ *films*^18^*, especially in the grains surface and interface, which can be effectively passivated by PCBM and C*_*60*_^6^,^19^*. In order to investigate the influence of different cathode materials (Ti, Au and PCBM) on the trap states, the corresponding photoluminescence (PL) emission spectra were measured and shown in* Fig. 3(a)*. Compared with the standard MAPbI*_*3*_ *sample spun on ITO glass, the Ti and PCBM films can cause blue-shift in the PL emitting wavelength of 12 and 10 eV respectively, while the Au film bring a blue-shift of about 4 eV. Based on the work of Huang’s group, the trap states can cause a red-shift of PL spectrum*^6^*. Therefore the PL results indicate that the Ti insertion layer can reduce the density of charge traps in the surface/interface, which is more effective than PCBM. On the contrary, the contact between MAPbI*_*3*_*and Au has brought more trap states than the standard sample.*

The voids at the interface between Au and MAPbI_3_ may induce accumulation of the charged ions or vacancies at the electrode interfaces, and then cause the ionic migration which may induce the photocurrent hysteresis in the ITO/PEDOT:PSS/MAPbI_3_/Au devices^8^,^20^. *The insertion of Ti layer can eliminate the voids and avoid the direct contact between Au and perovskite. Hence, the ionic migration can be ignored in the ITO/PEDOT:PSS/MAPbI*_*3*_*/Ti/Au devices. Moreover, the charge traps in MAPbI*_*3*_ *films, which can trap carriers and cause recombination*^6^,^19^*, are also responsible for the photocurrent hysteresis*^17^,^21^*. Our results have demonstrated that with a good contact, the Ti/Au film can also effectively reduce the density of charge traps at the interface between the cathode and MAPbI*_*3*_*. Therefore, there is no hysteresis in the ETM-free devices with ITO/PEDOT:PSS/ MAPbI*_*3*_*/Ti/Au structure, as show in* Fig. 1(h)*. Moreover, since the Ti insertion layers can eliminate the voids and traps, the interface morphology between the cathode and perovskite has a good reproducibility. Meanwhile, as the spin-coating/evaporation of ETMs is omitted, the fabrication factors that affect the devices performance are also reduced. Therefore, the ETM-free device with Ti/Au cathode has shown a high repeatability, as shown in* Fig. 1(d–g).

*Finally, we have investigated the stability of various device structures without encapsulation in an ambient environment at 30–20 °C and with ~50% humidity, as shown in* Fig. 3(b)*. (The time dependent photocurrent curves for each device are given in* Supplementary Figure S2*) The PCE of devices with PCBM/C*_*60*_*/Au and Au cathodes decays rapidly in air and almost drops to zero within 30 h. The degradation mechanisms occurring may be due to these reasons: (1) the absorption of oxygen/water by the PCBM*^22^*, (2) the acidic and hygroscopic properties of PEDOT:PSS*^23^,^24^ *and (3) incomplete coverage of the perovskite film by the Au cathode. In contrast, the device using the Ti/Au cathode discards the PCBM as a ETM and ensures a perfect contact between the perovskite and metal cathode, providing sufficient protection and avoiding the perovskite and PEDOT:PSS contact with the air. Moreover, it must be highlighted that Ti in direct contact with MAPbI*_*3*_ *showed no reaction for a long-term exposure to moisture and oxygen, what is different from other common metal cathodes, such as Au, Al, Ag and Cu*^13^*. These make the PCE of the Ti/Au based ETM-free device remain above 70% of the initial value even after 300 h of storage in the ambient environment.*

In conclusion, high-performance ETM-free PSCs using Ti/Au multilayer cathode have been demonstrated. The Ti insertion layer is a very simple and effective approach to increase the cell’s R_sh_, and improve the interface morphology between Au and perovskite, Moreover, our cells has also shown hysteresis-free photocurrent curve, high reproducibility and high stability in the ambient environment, due to the passivation of charge traps and the exclusion of voids at the interface. As a result, a high PCE of ~13% has been obtained and retain about 9.2% after 300 h storage in air at room temperature. This result not only reveals the promising applicability of Ti/Au multilayer in the ETM-free PSCs, but also affords a novel approach for high performance and decently stable PSCs.

## Methods

### Device fabrication and characterization

PEDOT:PSS (Clevious P VP AI 4083) was spin-coated on clean ITO substrates at a speed of 3000 revolutions per minute (r.p.m.) for 60 s, and then annealed at 120 °C for 30 min. The MAPbI_3_films (~350 nm) were fabricated by two-step spin-coating procedures in a nitrogen-filled glovebox (<0.1 ppm O_2_ and H_2_O). PbI_2_ (99%, Aldrich, 650 mg/ml in DMF) and MAI (synthesized with methylamine and hydroiodic acid^15^, 70 mg/ml in 2-propanol) were spun on PEDOT:PSS substrates at 6000 r.p.m. for 35 s respectively, followed by thermal annealing at 100 ^o^C for 1 h. Finally, the “ITO/PEDOT:PSS/MAPbI_3_/Ti/Au” devices were finished by thermal evaporation of metal cathode layers (Ti and Au) in turn, and “ITO/PEDOT:PSS MAPbI_3_/PCBM/C_60_/BCP/Au” devices were finished by spin coating of PCBM (99%, Nano-c, 20 mg/ml, 6000 r.p.m.) and thermal evaporation of C_60_ (30 nm) (99%, Nano-c), BCP (6 nm) (99%, Taiwan) and Au in turn.

## Additional Information

**How to cite this article**: Shi, T. *et al*. Ti/Au Cathode for Electronic transport material-free organic-inorganic hybrid perovskite solar cells. *Sci. Rep.* **6**, 39132; doi: 10.1038/srep39132 (2016).

**Publisher's note:** Springer Nature remains neutral with regard to jurisdictional claims in published maps and institutional affiliations.

## Supplementary Material

Supplementary Information

## Figures and Tables

**Figure 1:**
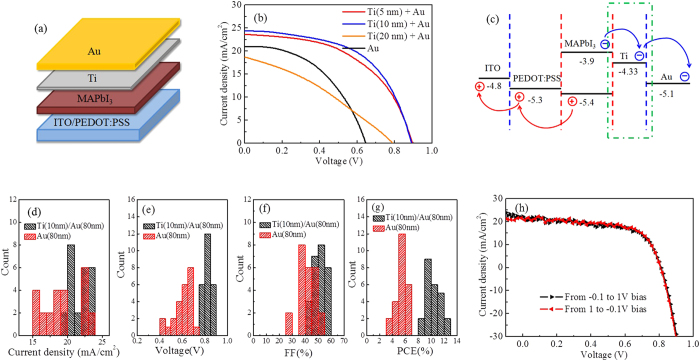
(**a**) Schematic drawing showing the vertical structure of the ITO/PEDOT:PSS/MAPbI_3_/Ti/Au devices; (**b**) J-V of the devices under 1.5 sun illumination with Au and Ti/Au cathodes, and with different Ti film thickness of 5, 10 and 20 nm; (**c**) Energy level diagram of the discussed solar cell which shows the charge separation process. The positions of the energy levels are according to ref. 10. (**d**–**g**) Histograms of short-circuit current density, open-circuit voltage, fill factor and power conversion efficiency of 24 cells for the devices with Ti(10 nm)/Au and Au cathode, respectively. (**h**) J-V curves with different scanning direction at a sweeping rate of 0.05 V/s under AM 1.5 G one sun illumination.

**Figure 2:**
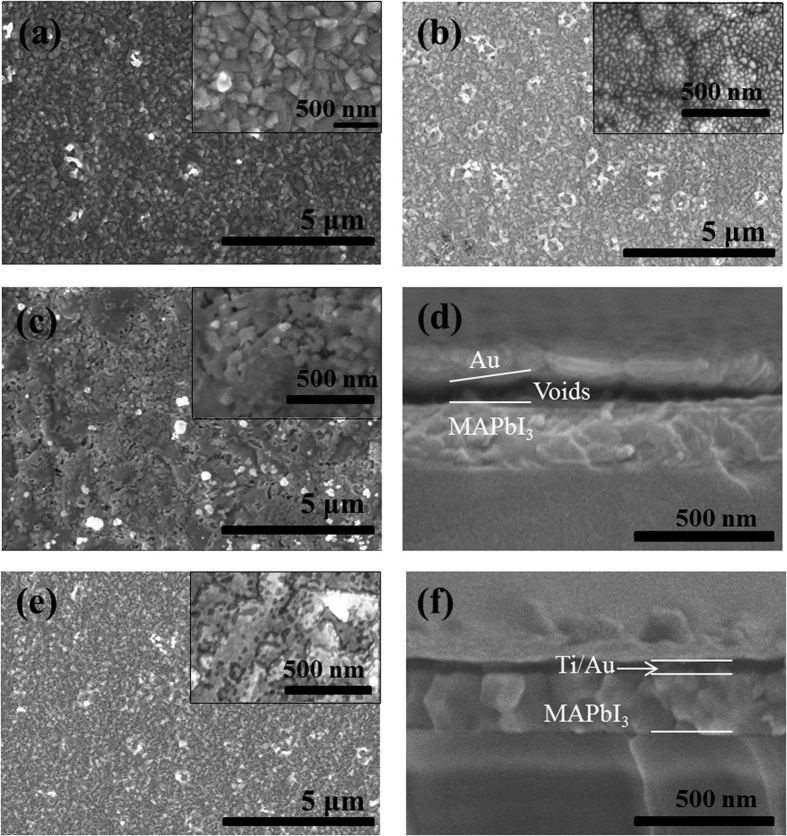
Top-view SEM images of the MAPbI_3_ (**a**), and different material layersf grown on MAPbI_3_ with different thickness: (**b**) Au (10 nm), (**c**) Au (20 nm) and (**e**) Ti (10 nm)/Au(10 nm), respectively. Cross-view SEM images of the ITO/PEDOT:PSS/MAPbI_3_/Au (**d**) and ITO/PEDOT:PSS/MAPbI_3_/Ti/Au (**f**), respectively.

**Figure 3:**
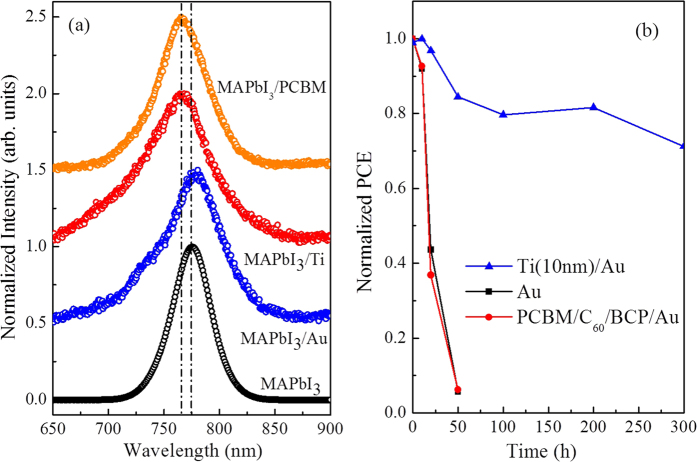
(**a**) Normalized PL emission spectra of the MAPbI_3_ on the surfaces of PCBM, Ti and Au at room temperature, respectively. (**b**) Normalized power conversion efficiency of perovskite solar cells with Ti/Au, Au and PCBM/C_60_/BCP/Au cathodes as a function of storage time in air.

**Table 1:** The best ETM-free solar cell photocurrent parameters based on different cathodes.

Sample	Jsc (mA/cm^2^)	Voc (V)	FF	PCE
Ti (5 nm)/Au	23.64	0.89	51.19%	10.77%
Ti (10 nm)/Au	24.38	0.89	59.49%	12.91%
Ti (20 nm)/Au	18.73	0.79	33.12%	4.90%
Au	22.47	0.65	43.11%	6.30%
