# Supplementary material for: Ti/Au Cathode for Electronic transport material-free organic-inorganic hybrid perovskite solar cells
Source: Sci Rep. 2016 Dec 20;6:39132. doi: 10.1038/srep39132 (PMC5171770; doi:10.1038/srep39132)
Supplement: Supplementary Information [file srep39132-s1.doc]

**Supporting Information**

**Ti/Au Cathode for Electronic transport material-free organic-inorganic** **hybrid perovskite solar cells**

Tongfei Shi*^1^, Jian Chen, Jianqiang Zheng^1^, Xinhua Li^1^, Bukang Zhou^1^, Huaxiang Cao^1^ & Yuqi Wang^1^

^1^Laboratory of Material Physics, Institute of Solid State Physics, Chinese Academy of Sciences, Hefei 230031, China

*Corresponding Author

E-mail address: [tfshi@issp.ac.cn](mailto:tfshi@issp.ac.cn)


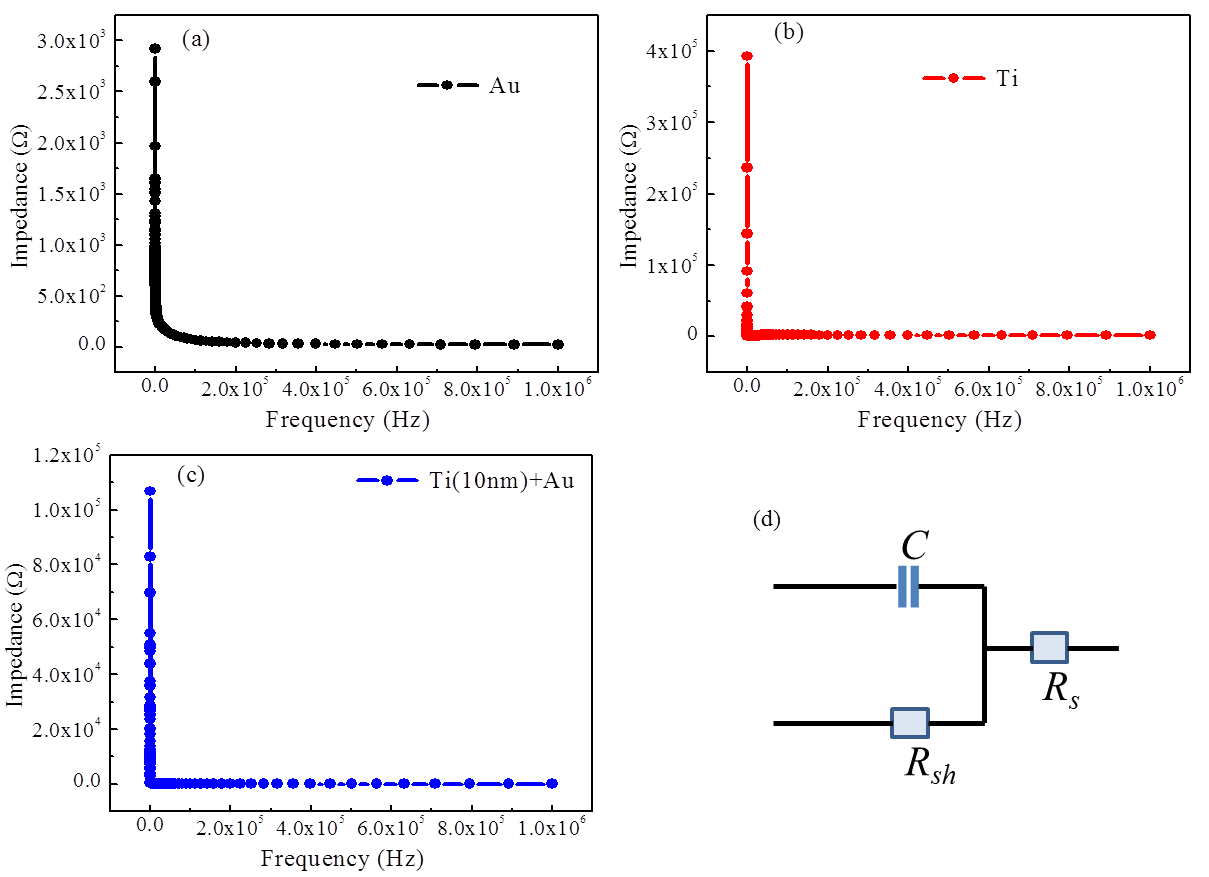


Figure S1 (a), (b) and (c) give electrochemical impedance spectroscopy (EIS) of the devices with Au (80 nm), Ti (60 nm) and Ti (10 nm)/Au (80 nm) cathodes, respectively. (d) Schematic of ideal model for single heterojunction solar cells.

We have measured electrochemical impedance spectroscopy (EIS) of the ETM-free devices with different cathodes (Au (80 nm), Ti (60 nm) and Ti (10 nm)/Au (80 nm)), and shown in Figure S1 (a), (b) and (c). Usually, there is an ideal model for single heterojunction solar cells, which is composed of a capacitor, a series resistance and a shunt resistance, as show in Figure S1 (d). According, the impendence of cells can be expressed by the following formula:

*R* = *R_s_* + 1/(j(ɷ*c*) + 1/*R_sh_*) (1)

where *R* is the impendence of the whole model, *R_s_* is the series resistance, *R_sh_* is the shunt resistance, ɷ is the frequency of the applied voltage, and *c* is the capacitance. When the ɷ approaches infinity or zero, the formula (1) can be simplified as the following formulas:

*R =* *R_s_* ɷ → ∞ (2)

*R = R_s_* + *R_sh_* ɷ → 0 (3)

Based on the formulas (2) and (3), we have obtained the value of *R_s_* and *R_sh_* of the devices with different cathodes form the EIS results, and shown in the following table.

| Sample | *R_s_* (Ω/cm^2^) | *R_sh_* (Ω/cm^2^) |
| --- | --- | --- |
| Au (80 nm) | 1.39 | 68.31 |
| Ti (10 nm)/Au (80 nm) | 8.94 | 6409.8 |
| Ti (60 nm) | 122.26 | 23558.4 |


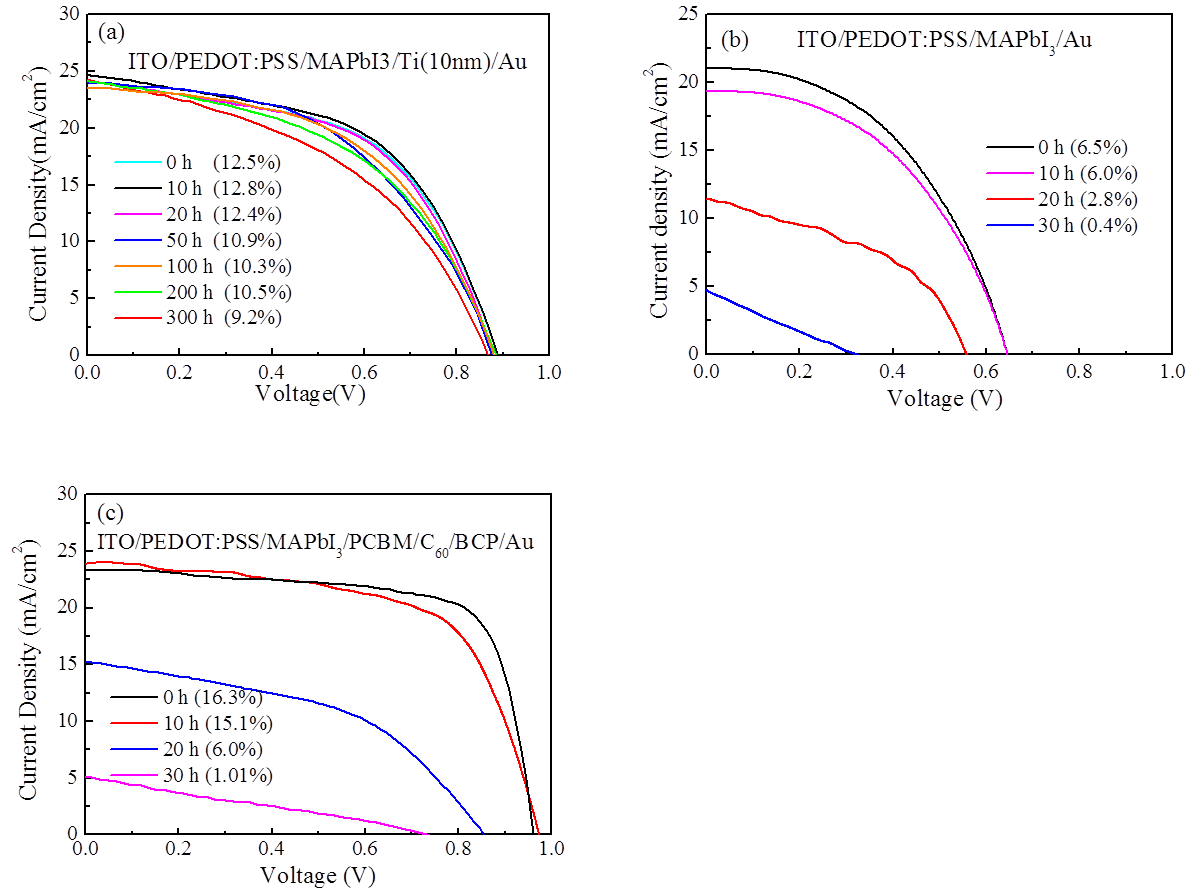


Figure S2. Time dependent *J-V* curves of the devices with Ti/Au (a), Au (b) and PCBM/C_60_/BCP/Au (c) cathodes, respectively. The PCE of each curve is indicated in the figure.
